# Pleiotropy of Cancer Susceptibility Variants on the Risk of Non-Hodgkin Lymphoma: The PAGE Consortium

**DOI:** 10.1371/journal.pone.0089791

**Published:** 2014-03-05

**Authors:** Unhee Lim, Jonathan M. Kocarnik, William S. Bush, Tara C. Matise, Christian Caberto, Sungshim Lani Park, Christopher S. Carlson, Ewa Deelman, David Duggan, Megan Fesinmeyer, Christopher A. Haiman, Brian E. Henderson, Lucia A. Hindorff, Laurence N. Kolonel, Ulrike Peters, Daniel O. Stram, Maarit Tiirikainen, Lynne R. Wilkens, Chunyuan Wu, Charles Kooperberg, Loïc Le Marchand

**Affiliations:** 1 Epidemiology Program, University of Hawaii Cancer Center, Honolulu, Hawaii, United States of America; 2 Public Health Sciences, Fred Hutchinson Cancer Research Center, Seattle, Washington, United States of America; 3 Center for Human Genetics Research, Vanderbilt University, Nashville, Tennessee, United States of America; 4 Department of Genetics, Rutgers University, Piscataway, New Jersey, United States of America; 5 Department of Preventive Medicine, Norris Comprehensive Cancer Center, Keck School of Medicine, University of Southern California, Los Angeles, California, United States of America; 6 Translational Genomics Research Institute, Phoenix, Arizona, United States of America; 7 Center for Child Health, Behavior and Development, Seattle Children's Research Institute, Seattle, Washington, United States of America; 8 Division of Genomic Medicine, National Human Genome Research Institute, National Institutes of Health, Bethesda, Maryland, United States of America; National Cancer Institute, National Institutes of Health, United States of America

## Abstract

**Background:**

Risk of non-Hodgkin lymphoma (NHL) is higher among individuals with a family history or a prior diagnosis of other cancers. Genome-wide association studies (GWAS) have suggested that some genetic susceptibility variants are associated with multiple complex traits (pleiotropy).

**Objective:**

We investigated whether common risk variants identified in cancer GWAS may also increase the risk of developing NHL as the first primary cancer.

**Methods:**

As part of the Population Architecture using Genomics and Epidemiology (PAGE) consortium, 113 cancer risk variants were analyzed in 1,441 NHL cases and 24,183 controls from three studies (BioVU, Multiethnic Cohort Study, Women's Health Initiative) for their association with the risk of overall NHL and common subtypes [diffuse large B-cell lymphoma (DLBCL), follicular lymphoma (FL), chronic lymphocytic leukemia or small lymphocytic lymphoma (CLL/SLL)] using an additive genetic model adjusted for age, sex and ethnicity. Study-specific results for each variant were meta-analyzed across studies.

**Results:**

The analysis of NHL subtype-specific GWAS SNPs and overall NHL suggested a shared genetic susceptibility between FL and DLBCL, particularly involving variants in the major histocompatibility complex region (rs6457327 in 6p21.33: FL OR = 1.29, *p* = 0.013; DLBCL OR = 1.23, *p* = 0.013; NHL OR = 1.22, *p* = 5.9×E-05). In the pleiotropy analysis, six risk variants for other cancers were associated with NHL risk, including variants for lung (rs401681 in *TERT*: OR per C allele = 0.89, *p* = 3.7×E-03; rs4975616 in *TERT*: OR per A allele = 0.90, *p* = 0.01; rs3131379 in *MSH5*: OR per T allele = 1.16, *p* = 0.03), prostate (rs7679673 in *TET2*: OR per C allele = 0.89, *p* = 5.7×E-03; rs10993994 in *MSMB*: OR per T allele = 1.09, *p* = 0.04), and breast (rs3817198 in *LSP1*: OR per C allele = 1.12, *p* = 0.01) cancers, but none of these associations remained significant after multiple test correction.

**Conclusion:**

This study does not support strong pleiotropic effects of non-NHL cancer risk variants in NHL etiology; however, larger studies are warranted.

## Introduction

Non-Hodgkin lymphoma (NHL) is the sixth most common incident cancer in the U.S. [1]. Although immune suppression, autoimmune disorders and certain infectious agents have been identified as strong risk factors for NHL, common host characteristics are also likely to be involved in the etiology of NHL [2]. Risk of NHL has been reported to be greater among individuals with a first-degree family history of hematopoietic cancers [3]. NHL is also a common second primary cancer among survivors of adult leukemia, laryngeal/pharyngeal cancer, renal cell carcinoma and melanoma, suggesting common genetic and/or environmental etiology, although it is difficult to rule out a treatment effect from the first cancer [4]–[6]. In searching for the shared genetic basis of disease, genome-wide association studies (GWAS) have discovered a number of risk variants that demonstrate associations with two or more complex traits (pleiotropy) [7]. A systematic review of the U.S. National Human Genome Research Institute (NHGRI) Catalog of Published GWAS reported that 16.9% of genes and 4.6% of single nucleotide polymorphisms (SNPs) in the catalog have shown such pleiotropic associations [8], [9]. The proportion of pleiotropic variants was higher than expected by chance and was particularly high among cancer risk variants, as well as among the variants associated with altered immunity and metabolic syndrome. Thus, genetic variations involved in cancer-related pathways may increase the risk of cancer of multiple types [10], including NHL. A good example is the multiple cancer site associations reported for variants at 8q24 [10], [11], a region where some lymphoid malignancies also exhibit translocations and a common susceptibility SNP [12]–[15].

In this study, we examined whether established risk variants identified in published GWAS of 17 common cancers present pleiotropic associations with the risk of NHL and its histologic subtypes in three well-characterized studies, as part of the Population Architecture using Genomics and Epidemiology (PAGE) consortium [16]. We also explored whether variants identified for specific NHL subtypes are also associated with overall NHL risk, and whether any such associations differ across ethnic groups.

## Materials and Methods

### Study Populations

The PAGE consortium was established in 2008 by the U.S. National Human Genome Research Institute to investigate well-replicated genetic variants for complex diseases in several large, ethnically diverse studies (https://www.pagestudy.org) [16]. Three PAGE studies participated in this analysis: biorepository of the Vanderbilt University (BioVU), the Multiethnic Cohort Study (MEC) and the Women's Health Initiative (WHI).

BioVU is a study at Vanderbilt University Medical Center that links de-identified electronic medical records (EMR) to a DNA biobank [17], [18]. Out of ∼130,000 BioVU participants, over 6,098 cancer cases were identified from 2009–2011 through linkage with the hospital tumor registry or search of diagnostic codes in the EMR. Race/ethnicity was recorded by hospital staff in the EMR (white, African American, Latino or Asian American) and confirmed using principal components analysis of ancestry-informative markers (AIMs). Controls included 9,152 BioVU patients without any prior or prevalent cancer diagnoses (except non-melanoma skin cancer) and with a similar reported race/ethnicity and age at clinic visit (within 5 years) as cancer cases. The MEC is a population-based prospective cohort of over 215,000 men and women in Hawaii and Los Angeles, aged 45–75 years at recruitment and primarily of five ancestries (white, African American, Latino, Japanese American or Native Hawaiian) [19], [20]. Incident cancer cases in the MEC were identified by linkage with Hawaii and California SEER tumor registries, from 1993 through October 2010. The WHI is a prospective cohort study investigating postmenopausal women's health in the U.S. [21]. A total of 161,838 women of ages 50–79 and of various race/ethnic groups (white, African American, Latino, Asian/Pacific Islander or American Indian) were recruited from 40 clinical centers throughout the U.S. in 1993–1998 for three clinical trials and an observational study. Medical history, including cancer incidence, is updated annually by mail and/or telephone questionnaires and confirmed by medical records and pathologic reports [22]. The current WHI analysis includes NHL cases identified through August 2009. All studies were approved by Institutional Review Boards at their respective study sites: the Vanderbilt Institutional Review Board for BioVU, the Human Studies Program at the University of Hawaii and Office for the Protection of Research Subjects at the University of Southern California for MEC, and the Fred Hutchinson Cancer Research Center Institutional Review Board for WHI. All participants of MEC and WHI provided written informed consent. All BioVU participants signed a “consent-to-treatment” form, informing them that anonymized genetic information from their discarded blood, along with de-identified EMR information, will be used for research and were given the choice to check an “opt-out” box if declining to participate [17].

### Selection of Cases and Controls

We limited our analysis to NHL cases and controls with no previous cancer (except non-melanoma skin cancer) in order to assess genetic pleiotropy without the possibility of confounding by previous cancers or treatments on the risk of NHL. MEC defined NHL cases based on the current World Health Organization classification that considered chronic lymphocytic leukemia (CLL) as a different presentation of the same disease as small lymphocytic lymphoma (SLL) [23], [24]. BioVU and WHI defined NHL based on the SEER classification and did not include CLL. Histology information based on the International Classification of Disease-Oncology (ICD-O3) was available in BioVU [25] and MEC [19] through linkage with tumor registries and in WHI [26] through systematic morphology coding of medical record information for the classification of the three most common NHL subtypes: diffuse large B-cell lymphoma (DLBCL; 9678–9680, 9684, 9689, 9699), follicular lymphoma (FL; 9690–9691, 9695, 9698) and CLL (9823)/SLL (9670) [24]. DLBCL was not ascertained in BioVU due to a prioritization for more common cancers in their PAGE analyses. For the current NHL analysis, all three studies included controls that were matched to cases of common cancers being investigated in the PAGE consortium (breast, colorectal, ovarian and prostate cancers and melanoma in all studies, and endometrial and lung cancers, and NHL in MEC and WHI). The matching was performed using frequency matching based on age at diagnosis or clinic visit (+/−5 yrs), sex and race/ethnicity in BioVU; and individual matching for each case based on age at cohort entry (+/−5 yrs), sex and race/ethnicity in MEC; and age at enrollment (+/−3 yrs), enrollment date (+/−365 days), race/ethnicity, and randomization arms (observational study or clinical trial assignment to hormone replacement therapy, dietary modification, or calcium/vitamin D supplement) in WHI. WHI also included additional controls selected from other genetic studies based on the availability of biomarkers.

### Biospecimen Collection, SNP Selection, and Genotyping

BioVU extracted DNA from discarded whole blood samples for patients drawn as part of routine clinical testing [17]. In the MEC, DNA of NHL cases was extracted from pre-diagnostic samples included in its prospective blood repository of over 67,000 cohort participants assembled in 2001–2006. DNA samples for controls were from either the prospective blood repository or from case-control studies of breast, colorectal and prostate cancers [20], [27]. The distribution of established cancer risk factors in the biospecimen sub-cohort was similar to that in the entire MEC cohort. DNA samples in WHI were extracted from pre-diagnostic blood collected at time of enrollment.

A total of 113 SNPs were selected and genotyped by one or more of the three PAGE studies based on genome-wide significant associations (*p*<5.0×E-08) [28] in the cancer GWAS literature at the time of the study design (March 2010). These non-NHL cancer SNPs included risk variants for bladder, brain (glioma), breast, colorectal, esophageal, lung, nasopharyngeal, neuroblastoma, ovarian, pancreatic, acute lymphoblastic leukemia, prostate, skin (basal cell carcinoma, melanoma), testicular germ cell, and thyroid cancers. The NHL SNPs, which included one risk variant for follicular lymphoma (FL) [29], [30] and 8 variants for chronic lymphocytic leukemia (CLL) [15], [30], [31], were only considered in associations with NHL and were excluded from the pleiotropy analysis. All samples were additionally genotyped for the ancestry informative markers (AIMs) described by Kosoy et al. [32] BioVU used Sequenom's iPLEX Gold coupled with MassARRAY MALDI-TOF MS detection and Illumina's BeadXpress with a custom GoldenGate genotyping assay. MEC used Applied Biosystems Taqman SNP genotyping assays on the OpenArray and the 7900HT Real-Time PCR platforms. WHI used Illumina BeadXpress with the Veracode GoldenGate genotyping assay. All sites used blind duplicate controls. Samples with low overall call rates (<90% of SNPs) were excluded. SNPs were excluded based on deviation from ethnicity-specific Hardy-Weinberg equilibrium (p<0.01), low call rates (<95%) or low concordance rates – range of minimum varied between 96.5 and 99% in the studies. In addition to site-specific quality control as above, all PAGE study sites genotyped the same 360 DNA samples from the International HapMap Project with excellent concordance rates with the published genotype data [16]. After these stringent quality control procedures, 1,441 NHL cases (BioVU, n = 293; MEC, n = 372; WHI, n = 776) and 24,183 controls (BioVU, n = 9,002; MEC, n = 9,091; WHI, n = 6,090) were included in the current analysis.

### Statistical Analysis

Unconditional logistic regression analysis was used in each study to estimate the association of cancer risk variants and NHL risk as odds ratios (ORs) and 95% confidence intervals (CIs). For each cancer risk variant, the allele that increased the risk of cancer in the original report was modeled against the low risk allele. Thus, ORs for NHL would be expected to be >1 if the association was in the same direction as the one found in the cancer GWAS study. Each biallelic SNP was coded as a continuous variable (0, 1 or 2 for number of risk alleles). The unconditional logistic regression model was adjusted for age, sex and race/ethnicity. Residual confounding by race/ethnicity was examined by additionally adjusting for principal components of genetic ancestry (top three in BioVU and WHI and top four in MEC). Effect modification by sex was assessed by a Wald test of the cross-product terms of sex and the continuous SNP variable in BioVU and MEC (WHI includes only women). Heterogeneity across race/ethnic groups was tested similarly using a Wald test in the MEC, where cases of non-white ethnic groups were available in substantial numbers. Also, heterogeneity in the SNP-cancer associations across common NHL subtypes (DLBCL, FL, CLL/SLL) was examined in the MEC, where all subtypes were ascertained, by performing polytomous logistic regression using common controls. A risk score was computed to examine the combined effect of 53 cancer variants that were genotyped in all three studies, by summing up the number of risk alleles (0, 1 or 2 for each SNP) in individuals across SNPs. For subjects with missing genotypes for any of the 53 variants, missing genotypes were estimated using the allele frequencies among controls of the same ethnicity in each study. The risk score was examined as both a continuous and a categorical variable (using quartile cut points based on the distribution among controls). To summarize results from the three studies, we carried out a meta-analysis for each variant and for the risk score variable in fixed-effects models using METAL [33]. Heterogeneity across studies was evaluated using Cochran's Q statistic. Analyses were conducted initially with significance considered at *p*<0.05 (two-sided). To control for the potentially inflated Type 1 error due to multiple comparisons, we used Bonferroni correction (*p* = 0.05/113 = 4.42E-04) to determine the statistical significance threshold for results.

## Results

Characteristics of the NHL cases and controls in the BioVU, MEC and WHI studies are shown in **Table 1**. Median age of NHL cases and controls was the highest in MEC, followed by WHI and BioVU, and both BioVU and MEC had a slightly higher representation of men over women. Cases in BioVU and WHI were mostly whites, whereas MEC had more even distribution of four ethnic groups.

**Table 1:** Characteristics of non-Hodgkin lymphoma (NHL) cases and controls in the PAGE studies.

	BioVU		MEC		WHI	
Type of Study	Cross-sectional [17], [18]		Nested Case-Control in Cohort [19], [20]		Nested Case-Control in Cohort [21], [22]	
Focus of Study	Cancer		Cancer		Women's health	
Years of Data Collection	Enrollment and Blood Draw 2007–2011; Diagnoses between 2009–2011		Enrollment 1993–1996; Blood draw 1995–2006; Diagnoses between 1993 and October 2010		Enrollment and Blood Draw 1993–1998; Diagnoses between 1993 and August 2009	
	NHL Cases	Controls	NHL Cases	Controls	NHL Cases	Controls
Selection	First primary incident NHL diagnoses from hospital tumor registry and electronic medical records (EMR)	Combined controls for multiple cancer sites; matched on age, sex, ethnicity	First primary incident NHL diagnoses from linkage of cohort with SEER* registries	Combined controls for multiple cancer sites; matched on age, sex, ethnicity	First primary incident NHL diagnoses from active follow-up (semi/annual) and EMR verification	Combined controls for multiple cancer sites; matched on age, enrollment date, ethnicity, randomization
Total, n*	293	9,002	372	9,091	776	6,090
Age, median (range)	57 (18–102)	63 (19–110)	71 (45–92)	71 (45–88)	65 (50–79)	65 (50–79)
Sex, n (%) women	139 (47%)	3,711 (41%)	166 (45%)	4,321 (48%)	776 (100%)	6,090 (100%)
Race/Ethnicity, n (%)						
White	275 (94%)	8,061 (90%)	102 (27%)	1844 (20%)	718 (93%)	4,763 (78%)
African American	16 (5%)	804 (9%)	68 (18%)	2228 (25%)	27 (3%)	714 (12%)
Latino	0	56 (0.6%)	80 (22%)	1864 (21%)	16 (2%)	332 (5%)
Asian American/Pacific Islander	2/0 (0.7%)	81/0 (0.9%)	104/18 (33%)	2513/642 (35%)	15/0 (2%)	281/0 (5%)
NHL Subtypes, n (%)	293	N/A	372	N/A	776	N/A
DLBCL	-		102 (27%)		258 (33%)	
FL	72 (25%)		68 (18%)		178 (23%)	
CLL/SLL	42 (SLL only; 14%)		71 (19%)		66 (SLL only; 9%)	
Others	179 (61%)		131 (35%)		274 (35%)	

* Any prior cancer cases were excluded from the NHL cases and controls for the current analysis, based on self-report (BioVU, MEC, WHI), the SEER registry linkage (BioVU, MEC), and medical record reviews (BioVU, WHI).

Abbreviations: BioVU (the biorepository of the Vanderbilt University), MEC (the Multiethnic Cohort Study), WHI (the Women's Health Initiative); CLL/SLL (chronic lymphocytic leukemia/small lymphocytic lymphoma), DLBCL (diffuse large B-cell lymphoma), FL (follicular lymphoma), SEER (Surveillance, Epidemiology and End Results).

**Figure 1:**
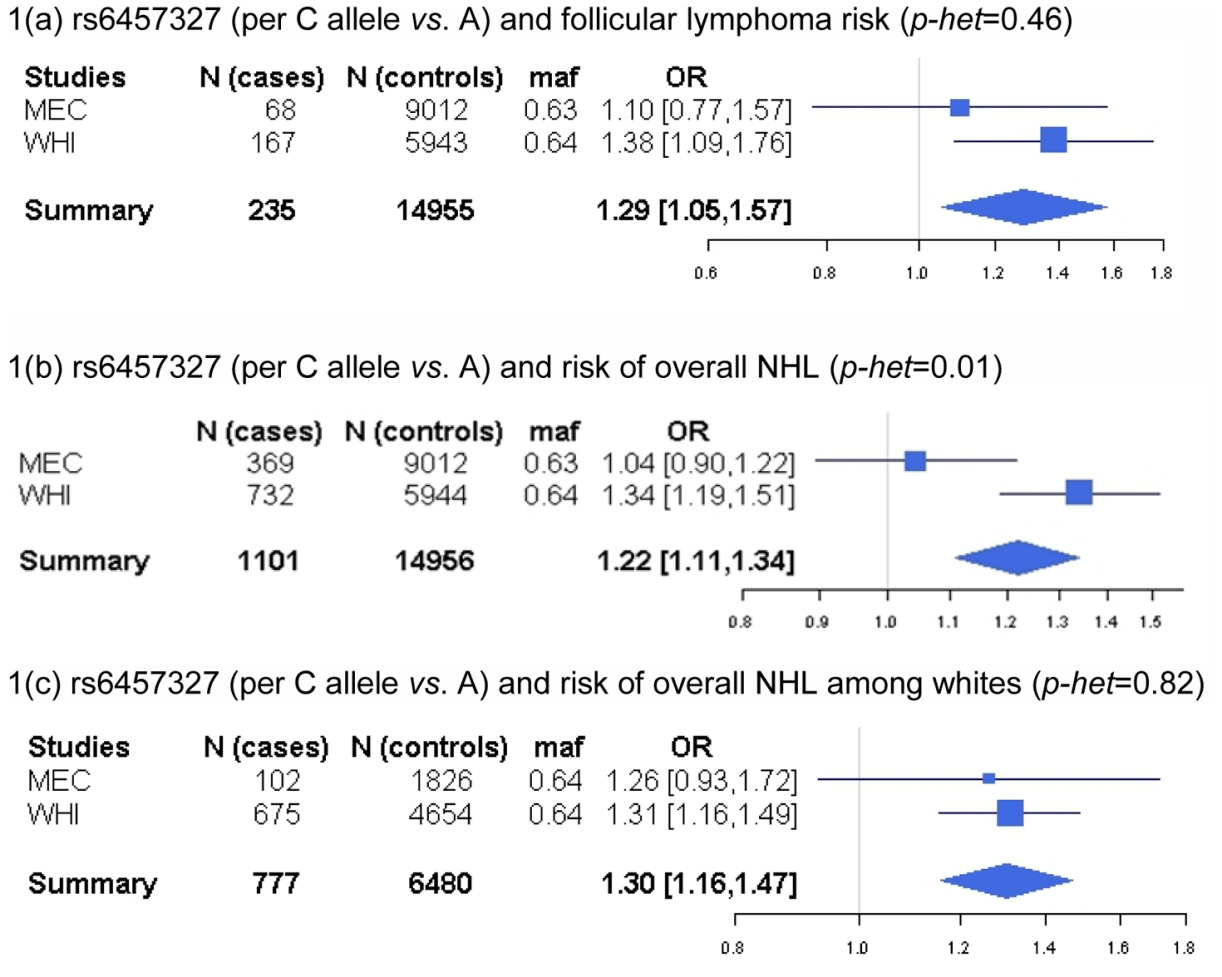
Forest plots for the association between a published follicular lymphoma risk variant (rs6457327) and the risk of follicular lymphoma or overall non-Hodgkin lymphoma (NHL) in the Multiethnic Cohort (MEC) and the Women's Health Initiative (WHI) in the PAGE consortium. (a) follicular lymphoma, (b) overall NHL, and (c) overall NHL among whites only.

We first investigated nine previously published GWAS risk variants for specific NHL subtypes (one for FL and 8 for CLL) for an association with overall NHL risk to test for a shared genetic susceptibility. The association reported for FL with rs6457327, in 6p21.33, the major histocompatibility complex region (MHC), replicated in our data for FL [summary OR per allele C *vs.* A = 1.29 (1.05–1.57), *p* = 0.013; **Figure 1(a)**, **Table S1 in File S1**] and was also observed for DLBCL [OR = 1.23 (1.04–1.44), *p* = 0.013] and overall NHL [OR = 1.22 (1.11–1.34), *p* = 5.92E-05; **Figure 1(b)**], but not for CLL/SLL [OR = 1.05 (0.81–1.36), *p* = 0.73]. When the meta-analysis on overall NHL was limited to MEC and WHI, considering that BioVU did not include DLBCL, the association with the published FL risk variant remained the same [OR = 1.22 (1.11–1.33), *p* = 5.92E-05]. In the MEC, where all main subtypes were examined, including CLL, the OR for the association of allele C of rs6457327 with FL did not differ from the corresponding OR for DLBCL (*p-het.* from polytomous regression = 0.61), CLL/SLL (*p-het*. = 0.23) or other subtypes (*p-het*. = 0.73). We did observe heterogeneity in the association for the FL risk variant (rs6457327) and overall NHL across study sites (Cochran Q = 6.58; *p-het*. = 0.01), which was eliminated when the analysis was limited to whites only [**Figure 1(c)**; OR for allele C of rs6457327 = 1.30 (1.16–1.47), *p* = 8.83E-06; Cochran Q = 0.05, *p-het*. = 0.82], although the interaction between the variant and race/ethnicity was not significant in the MEC (*p-int*. = 0.10). Among the eight GWAS risk variants for CLL, one variant replicated in our data for CLL/SLL [OR per allele G of rs17483466 in *ACOXL/BCL2L11* = 1.57 (1.17–2.11), *p* = 0.0027] and remained significant after Bonferroni correction (*p*<0.0063; Table S1 in File S1). None of the CLL variants were associated with overall NHL risk (*p*>0.05; data not shown). For example, in the MEC, the association of rs17483466 with CLL/SLL significantly differed from that with FL (*p-het*. from polytomous regression = 0.03), DLBCL (*p-het*. = 0.02) and others (*p-het*. = 0.01).

Of the 113 GWAS risk variants for cancers other than NHL that were examined in PAGE, 53 SNPs were genotyped in all three studies, and the other 60 variants were typed in one or two studies (**Table S2 in File S1**). Six of the 53 SNPs showed nominal associations with the risk of overall NHL, including three risk variants originally identified for lung cancer, two risk variants identified for prostate cancer and one risk variant for breast cancer (**Table 2**). None of these associations remained significant after multiple test correction (i.e., *p*>4.4E-04 for 113 SNPs). Two lung cancer risk variants in the *TERT* region (rs401681, OR per allele C = 0.89 (0.82–0.96), *p* = 0.0037; rs4975616, OR per allele A = 0.90 (0.83–0.97), *p* = 0.010), as well as the prostate cancer risk variant (rs7679673 in *TET2*; OR per allele C = 0.89 (0.82–0.97), *p* = 0.0057), were associated with a decreased risk of overall NHL. The two *TERT* variants were in linkage disequilibrium (LD) among whites (R^2^ = 0.84) and Native Hawaiians (R^2^ = 0.79) but less so in other ethnic groups (R^2^ = 0.53 for African Americans; 0.47 for Latinos, 0.30 for Japanese Americans). The breast cancer susceptibility variant rs3817198 in *LSP1* [OR per allele C = 1.12 (1.03–1.22), *p* = 0.011], the lung cancer SNP rs3131379 in the MHC region in chromosome 6 (6p21.33) [OR per allele T = 1.16 (1.01–1.33), *p* = 0.030] and the prostate cancer variant rs10993994 in *MSMB* [OR per allele T = 1.09 (1.01–1.18), *p* = 0.036] were each associated with an increased risk of overall NHL. The associations for the six variants above did not differ significantly across study sites, except for rs401681 (*TERT*), which showed a stronger inverse association in BioVU and MEC than in WHI (Cochran Q = 6.26, *p-het.* = 0.04; Table 2). These 6 variants showed the same or similar summary ORs when the analysis was limited to MEC and WHI, where overall NHL included DLBCL, with 4 variants showing nominal significance (unadjusted *p*<0.05; data not shown). Of the other 60 variants genotyped in only two studies or a single study, 7 SNPs showed moderate associations (unadjusted *p*<0.05; data not shown). In particular, an esophageal cancer variant (rs1229984 in *ADH1B*) available in MEC and WHI showed an inverse association with NHL risk [OR per allele C = 0.77 (0.66–0.90), *p* = 4.4E-04].

**Table 2:** Pleiotropic association of selected cancer susceptibility variants with the risk of overall non-Hodgkin lymphoma (NHL).

				BioVU		MEC		WHI		Summary		
SNP	Gene	GWAS	Risk (Ref.) Allele	Cases/Controls	OR (95% CI)*	Cases/Controls	OR (95% CI)*	Cases/Controls	OR (95% CI)*	OR (95% CI)*	*p-value* (0.00044)†	Cochran Q (*p-het*)
rs401681	*TERT*	Lung	C (T)	292/8984	0.88 (0.74, 1.04)	372/9053	0.76 (0.66, 0.89)	733/5939	0.97 (0.87, 1.09)	0.89 (0.82, 0.96)	0.0037	6.26 (0.04)
rs7679673	*TET2*	Prostate	C (A)	290/8899	1.02 (0.86, 1.22)	372/8946	0.88 (0.75, 1.04)	733/5936	0.84 (0.75, 0.94)	0.89 (0.82, 0.97)	0.0057	3.42 (0.18)
rs4975616	*TERT*	Lung	A (G)	293/9000	0.85 (0.72, 1.00)	368/9058	0.80 (0.68, 0.95)	732/5938	0.97 (0.87, 1.09)	0.90 (0.83, 0.97)	0.0103	4.06 (0.13)
rs3817198	*LSP1*	Breast	C (T)	293/8995	1.06 (0.89, 1.27)	371/9042	1.11 (0.93, 1.32)	733/5942	1.15 (1.02, 1.30)	1.12 (1.03, 1.22)	0.0112	0.56 (0.76)
rs3131379	*MSH5*	Lung	T (C)	290/8916	1.08 (0.83, 1.41)	334/9053	1.11 (0.78, 1.59)	733/5946	1.21 (1.01, 1.43)	1.16 (1.01, 1.33)	0.0302	0.51 (0.78)
rs10993994	*MSMB*	Prostate	T (C)	292/9001	1.20 (1.01, 1.42)	369/9054	1.08 (0.93, 1.26)	732/5944	1.05 (0.94, 1.17)	1.09 (1.01, 1.18)	0.0356	1.68 (0.43)

* ORs and 95% CIs in individual studies were estimated in unconditional logistic regression models that were adjusted for age, sex (in BioVU and MEC) and ethnicity (ancestry informative markers). Summary ORs and 95% CIs were estimated in a meta-analysis of fixed-effects models.

† The Bonferroni corrected *p-value* for 53 SNPs/tests is 4.4E-04.

Abbreviations: *p*-het. (*P*-values for heterogeneity across studies measured in Cochran's Q statistic); BioVU (the biorepository of the Vanderbilt University), MEC (the Multiethnic Cohort Study), WHI (the Women's Health Initiative).

The pleiotropy analysis for specific NHL subtypes was conducted on all non-NHL GWAS cancer SNPs (n = 113) (**Table S3 in File S1**). None of the subtype-specific associations were significant after Bonferroni correction (i.e., *p*>0.05/113 = 4.4E-04 for 113 tests on each subtype). The most significant association for follicular lymphoma was with a breast cancer risk variant [rs11249433 in *EMBP1*: summary OR per allele C = 1.29 (1.08–1.54), *p* = 0.0095]. For CLL/SLL, the most significant association was with a prostate cancer risk variant [rs2735839 in *KLK3-KLK2*: OR per allele G = 1.51 (1.10–2.07), *p* = 0.0099]. The associations of overall NHL described above (Table 2) with the risk variants for lung cancer (rs3131379 in *MSH5*) and prostate cancer (rs7679673 in *TET2*) appeared to be due to their associations with the risk of DLBCL subtype [OR per allele T in rs3131379 = 1.41 (1.10–1.80), *p* = 0.0061; OR per allele C in rs7679673 = 0.83 (0.71–0.98), *p* = 0.030].

The risk score based on the 53 non-NHL cancer SNPs was not significantly associated with the risk of overall NHL or subtypes, either as a continuous variable (**Table 3**) or categorized in quartiles (*p*>0.05; data not shown). There was no significant heterogeneity in any of the associations for individual SNPs or the risk score by sex, or by ethnic group (*p*>0.05; data not shown).

**Table 3:** Associations between a risk score (RS) for 53 GWAS-identified cancer risk variants and the overall and subtype-specific risks of NHL.

	BioVU		MEC		WHI		Summary			
	Mean RS, case/control	OR (95% CI)*	Mean RS, case/control	OR (95% CI)*	Mean RS, case/control	OR (95% CI)*	N, case/control	OR (95% CI)*	*p-value*	Cochran Q (*p-het*)
Overall NHL	47.4/46.9	0.98 (0.96, 1.01)	44.1/44.2	0.98 (0.94, 1.02)	42.4/42.4	1.00 (0.98, 1.01)	1,414/23,469	1.00 (0.98, 1.01)	0.80	3.27 (0.20)
DLBCL	-	-	43.5/44.2	0.98 (0.94, 1.02)	42.8/42.4	1.01 (0.99, 1.04)	360/23,469	1.00 (0.98, 1.03)	0.79	2.00 (0.16)
FL	47.0/46.9	1.00 (0.96, 1.05)	44.7/44.2	1.03 (0.98, 1.09)	42.9/42.4	1.02 (0.98, 1.05)	318/23,469	1.02 (0.99, 1.04)	0.17	0.85 (0.65)
CLL/SLL	48.1/46.9	1.05 (0.98, 1.12)	46.2/44.2	1.04 (0.99, 1.09)	42.2/42.4	0.98 (0.93, 1.04)	179/23,469	1.02 (0.99, 1.05)	0.19	2.75 (0.25)

* ORs and 95% CIs in individual studies were estimated per risk allele in unconditional logistic regression models that were adjusted for age, sex (in BioVU and MEC) and ethnicity. Summary odds ratios (ORs) and 95% confidence intervals (CIs) were estimated in a meta-analysis of fixed effects models.

Abbreviations: *p-het*. (*p-values* for heterogeneity across studies measured in Cochran's Q statistic); BioVU (the biorepository of Vanderbilt University), MEC (the Multiethnic Cohort Study), WHI (the Women's Health Initiative).

## Discussion

Increasing evidence supports the pleiotropic involvement of common genetic risk variants in multiple diseases or complex traits. Thus, we examined a substantial number of risk variants identified in GWAS of common cancers in relation to overall and subtype-specific risk of NHL. Our analysis extended the association of the FL risk variant to overall NHL and the DLBCL subtype specifically, as indicated in previous studies for shared etiology [34]–[36]. The CLL risk variants did not extend to other subtypes or NHL overall, indicating the subtype-specificity of the CLL variants. For non-NHL cancer variants, we found no convincing evidence of pleiotropy, with only weak suggestions that specific risk variants for lung, prostate and breast cancers may also be associated with the risk of developing first primary incident NHL among those without a history of other cancers or prior cancer treatments.

The effects of three of the six non-NHL GWAS variants nominally associated with NHL (rs401681, rs7679673, rs4975616) were in the opposite direction compared to the original reports. These variants showed an association with increased risks for lung and prostate cancers in the original reports but a lower risk of NHL in our study. This may be a chance finding given that none of the associations remained significant after correcting for multiple tests, though such effects in opposite directions for different cancer types have been previously demonstrated in the *TERT* region and SNP rs401681 in particular [37]. A prostate cancer variant (rs7679673 in *TET2*) was specifically associated with a lower risk of DLBCL.

One of the other three nominally positive associations for overall NHL was found with a breast cancer SNP in the coding region for a lymphocyte-specific protein (rs3817198 in *LSP1*). This gene encodes an intracellular F-actin binding protein that is expressed in endothelium and various hematopoietic cells (lymphocytes, neutrophils, macrophages) [38]. As such, this protein may be involved in lymphomagenesis through the regulation of lymphocyte motility and migration, as evidenced by an association of another variant in *LSP1* (rs2089910) with NHL in a study of an immune and inflammation SNP panel [39].

Another non-significant positive association for NHL, especially with DLBCL, was with a lung cancer susceptibility variant, rs3131379, in *MSH5* or *mutS homolog 5*, a gene involved in the DNA mismatch repair pathway [40]. This variant is also located near the major histocompatibility complex (MHC or human leukocyte antigen, HLA) region in chromosome 6 (6p21.33), as is the GWAS variant for FL (rs6457327), and has been associated with the risk of systemic lupus erythematosus in a GWAS [41]. Our findings on *MSH5* and *MHC* variants indicate possible involvement of variants in or near this highly-conserved immune-regulatory region in the etiology of NHL (including FL and DLBCL), in addition to that of lung cancer.

This study was nested in three large studies with well-characterized phenotypes and pathology-confirmed histologic information for subtype classification. However, despite the sizeable number of NHL cases included, we had limited power, in part likely due to the heterogeneous nature of NHL. Also, only a subset of total cancer variants was genotyped in all three PAGE studies for the NHL analysis. Our analyses do not provide clear evidence that these common cancer genetic susceptibility loci may play a role in the etiologies of NHL. A more systematic approach in larger pooled analyses of specific subtypes, with larger SNP panels, is warranted in future research.

## Supporting Information

Files S1 **Supporting tables. Table S1.** Association between established GWAS risk variants for follicular lymphoma (FL) and for chronic lymphocytic leukemia (CLL) with the risk of these subtypes of non-Hodgkin lymphoma (NHL). **Table S2.** List of 113 GWAS-based cancer risk variants examined for pleiotropy on NHL in PAGE; the 53 SNPs listed as genotyped in all three studies were included in the risk score analysis. **Table S3.** Pleiotropic association of selected cancer susceptibility variants with the risk of common subtypes of non-Hodgkin lymphoma (NHL).
(DOC)
