# Supplementary material for: Pleiotropy of Cancer Susceptibility Variants on the Risk of Non-Hodgkin Lymphoma: The PAGE Consortium
Source: PLoS One. 2014 Mar 5;9(3):e89791. doi: 10.1371/journal.pone.0089791 (PMC3943855; doi:10.1371/journal.pone.0089791)
Supplement: Files S1 — Supporting tables. Table S1. Association between established GWAS risk variants for follicular lymphoma (FL) and for chronic lymphocytic leukemia (CLL) with the risk of these subtypes of non-Hodgkin lymphoma (NHL). Table S2. List of 113 GWAS-based cancer risk variants examined for pleiotropy on NHL in PAGE; the 53 SNPs listed as genotyped in all three studies were included in the risk score analysis. Table S3. Pleiotropic association of selected cancer susceptibility variants with the risk of common subtypes of non-Hodgkin lymphoma (NHL). (DOC) [file pone.0089791.s001.doc]

Table S1. Association between established GWAS risk variants for follicular lymphoma (FL) and for chronic lymphocytic leukemia (CLL) with the risk of these subtypes of non-Hodgkin lymphoma (NHL)

| Subtype  / SNP | Gene | Location | Risk (Ref.) Allele | MEC | | WHI | | Summary (meta-analysis) | | |
| --- | --- | --- | --- | --- | --- | --- | --- | --- | --- | --- |
|  |  |  |  | Cases /  Controls | OR (95% CI)* | Cases /  Controls | OR (95% CI)* | OR (95% CI)* | *p-value*† | Cochran Q  (*p-het*) |
| Follicular Lymphoma (FL) risk variant and its association with FL risk | | | | | | | | | | |
| rs6457327 | *intergenic (HCG22-C6orf15, STG, PSORS1)* | 6p21.33 | C (A) | 68 / 9012 | 1.10 (0.77, 1.57) | 167 / 5943 | 1.38 (1.09, 1.76) | 1.29 (1.05, 1.57) | 0.01290 | 1.07 (0.30) |
| Chronic Lymphocytic Leukemia (CLL) / Small Lymphocytic Lymphoma (SLL) risk variants and their associations with CLL/SLL risk | | | | | | | | | | |
| rs11083846 | *PRKD2* | 19q13.32 | A (G) | 68 / 9021 | 1.32 (0.84, 2.08) | 61 / 5940 | 1.12 (0.73, 1.71) | 1.21 (0.89, 1.65) | 0.23270 | 0.26 (0.61) |
| rs13397985 | *SP140/SP110* | 2q37.1 | G (T) | 69 / 9010 | 1.60 (1.03, 2.49) | 61 / 5926 | 1.07 (0.67, 1.69) | 1.32 (0.96, 1.82) | 0.08601 | 1.57 (0.21) |
| rs17483466 | *ACOXL/BCL2L11* | 2q13 | G (A) | 69 / 8999 | 1.76 (1.16, 2.69) | 61 / 5939 | 1.41 (0.93, 2.12) | 1.57 (1.17, 2.11) | **0.00266** | 0.56 (0.45) |
| rs2456449 | *8q24.21* | 8q24.21 | G (A) | - | - | 60 / 5929 | 1.16 (0.80, 1.69) | - | - | - |
| rs7176508  (rs10220831) | *RPLP1* | 15q23 | A (G) | (excluded in QC process) | | 61 / 5940 | 1.17 (0.81, 1.69) | - | - | - |
| rs735665 | *intergenic (ASAM-GRAMD18)* | 11q24.1 | A (G) | 68 / 8999 | 1.49 (0.94, 2.36) | 61 / 5939 | 1.18 (0.76, 1.84) | 1.32 (0.96, 1.82) | 0.08843 | 0.51 (0.48) |
| rs757978 | *FARP2* | 2q37.3 | A (G) | - | - | 61 / 5944 | 0.67 (0.34, 1.33) | - | - | - |
| rs872071 | *IRF4* | 6p25.3 | G (A) | 69 / 9005 | 1.19 (0.84, 1.71) | 60 / 5938 | 1.21 (0.83, 1.74) | 1.20 (0.93, 1.56) | 0.16310 | 0.001 (0.97) |

* ORs and 95% CIs in individual studies were estimated in unconditional logistic regression models that were adjusted for age, sex (MEC only) and ethnicity. Summary ORs and 95% CIs were estimated in a meta-analysis of fixed effects models.

† The *p-value* corrected for testing 8 SNPs on CLL/SLL is 0.0063.

Abbreviations: p-het. (p-values for heterogeneity across studies measured in Cochran’s Q statistic); MEC (the Multiethnic Cohort Study), WHI (the Women’s Health Initiative). GWAS risk variants for FL and CLL were not typed in BioVU.

Table S2. List of 113 GWAS-based cancer risk variants examined for pleiotropy on NHL in PAGE; the 53 SNPs listed as genotyped in all three studies were included in the risk score analysis.

| SNP ID | Nearest Gene | Locus | GWAS Cancer | Alleles | | PAGE Studies |
| --- | --- | --- | --- | --- | --- | --- |
|  |  |  |  | Risk | Referent |  |
| ***Genotyped in three studies (n=53)*** | | | | | | |
| RS11249433 | *EMBP1* | 1p11.2 | breast | C | T | BioVU, MEC, WHI |
| RS1465618 | *THADA* | 2p21 | prostate | T | C | BioVU, MEC, WHI |
| RS12621278 | *ITGA6* | 2q31.1 | prostate | A | G | BioVU, MEC, WHI |
| RS13387042 | *intergenic (TNP1-DIRC3)* | 2q35 | breast | A | G | BioVU, MEC, WHI |
| RS2660753 | *intergenic (VGLL3-CHMP2B)* | 3p12.1 | prostate | T | C | BioVU, MEC, WHI |
| RS4973768 | *SLC4A7* | 3p24.1 | breast | T | C | BioVU, MEC, WHI |
| RS4857841 | *EFFSEC* | 3q21.3 | prostate | A | G | BioVU, MEC, WHI |
| RS7626795 | *IL1RAP* | 3q28 | lung | G | A | BioVU, MEC, WHI |
| RS12500426 | *PDLIM5* | 4q22.3 | prostate | A | C | BioVU, MEC, WHI |
| RS17021918 | *PDLIM5* | 4q22.3 | prostate | C | T | BioVU, MEC, WHI |
| RS7679673 | *intergenic (RPL6P14-TET2)* | 4q24 | prostate | C | A | BioVU, MEC, WHI |
| RS2736100 | *TERT* | 5p15.33 | glioma | G | T | BioVU, MEC, WHI |
| RS4975616 | *intergenic (TERT-CLPTM1L)* | 5p15.33 | lung | A | G | BioVU, MEC, WHI |
| RS401681 | *TERT, CLPTM1L* | 5p15.33 | lung | C | T | BioVU, MEC, WHI |
| RS889312 | *intergenic (RPL26P19-MAP3K1)* | 5q11.2 | breast | C | A | BioVU, MEC, WHI |
| RS3131379 | *MSH5* | 6p21.33 | lung | T | C | BioVU, MEC, WHI |
| RS2046210 | *intergenic (ESR1-C6orf97)* | 6q25.1 | breast | A | G | BioVU, MEC, WHI |
| RS9364554 | *SLC22A3* | 6q25.3 | prostate | T | C | BioVU, MEC, WHI |
| RS10486567 | *JAZF1* | 7p15.2 | prostate | G | A | BioVU, MEC, WHI |
| RS6465657 | *LMTK2* | 7q21.3 | prostate | C | T | BioVU, MEC, WHI |
| RS2928679 | *intergenic (SLC25A37)* | 8p21 | prostate | A | G | BioVU, MEC, WHI |
| RS1512268 | *intergenic (SLC25A37-NKX3.1)* | 8p21.2 | prostate | T | C | BioVU, MEC, WHI |
| RS16892766 | *intergenic (TRPS1-EIF3H)* | 8q23.3 | colorectal | C | A | BioVU, MEC, WHI |
| RS13281615 | *intergenic (SRRM1P1)* | 8q24.21 | breast | T | C | BioVU, MEC, WHI |
| RS6983267 | *intergenic (SRRM1P1-POU5F1B)* | 8q24.21 | colorectal | G | T | BioVU, MEC, WHI |
| RS7014346 | *intergenic (SRRM1P1-POU5F1B)* | 8q24.21 | colorectal | A | G | BioVU, MEC, WHI |
| RS4636294 | *intergenic (IFNE-MTAP)* | 9p21.3 | skin (melanoma) | A | G | BioVU, MEC, WHI |
| RS3814113 | *intergenic (BNC2-RPL31P42)* | 9p22.2 | ovarian | T | C | BioVU, MEC, WHI |
| RS10795668 | *intergenic (KRT8P16-TCEB1P3)* | 10p14 | colorectal | G | A | BioVU, MEC, WHI |
| RS10993994 | *MSMB* | 10q11.23 | prostate | T | C | BioVU, MEC, WHI |
| RS3817198 | *LSP1* | 11p15.5 | breast | C | T | BioVU, MEC, WHI |
| RS12418451 | *intergenic (TPCN2)* | 11q13 | prostate | A | G | BioVU, MEC, WHI |
| RS10896449 | *intergenic (TPCN2-MYEOV)* | 11q13.3 | prostate | G | A | BioVU, MEC, WHI |
| RS1393350 | *TYR* | 11q14.3 | skin (melanoma) | A | G | BioVU, MEC, WHI |
| RS3802842 | *C11orf93* | 11q23.1 | colorectal | C | A | BioVU, MEC, WHI |
| RS4444235 | *intergenic (RPS3AP46-BMP4)* | 14q22.2 | colorectal | C | T | BioVU, MEC, WHI |
| RS1051730 | *CHRNA3* | 15q25.1 | lung | A | G | BioVU, MEC, WHI |
| RS8042374 | *CHRNA3* | 15q25.1 | lung | G | A | BioVU, MEC, WHI |
| RS3803662 | *intergenic (TOX3-CHD9)* | 16q12.1 | breast | T | C | BioVU, MEC, WHI |
| RS258322 | *CDK10* | 16q24.3 | skin (melanoma) | A | G | BioVU, MEC, WHI |
| RS4785763 | *AFG3L1* | 16q24.3 | skin (melanoma) | A | C | BioVU, MEC, WHI |
| RS11649743 | *HNF1B* | 17q12 | prostate | G | A | BioVU, MEC, WHI |
| RS4430796 | *HNF1B* | 17q12 | prostate | G | A | BioVU, MEC, WHI |
| RS7501939 | *HNF1B* | 17q12 | prostate | C | T | BioVU, MEC, WHI |
| RS4939827 | *SMAD7* | 18q21.1 | colorectal | T | C | BioVU, MEC, WHI |
| RS10411210 | *RHPN2* | 19q13.11 | colorectal | C | T | BioVU, MEC, WHI |
| RS266849 | *intergenic (KLK15-KLK3)* | 19q13.33 | prostate | A | G | BioVU, MEC, WHI |
| RS2735839 | *intergenic (KLK3-KLK2)* | 19q13.33 | prostate | G | A | BioVU, MEC, WHI |
| RS961253 | *intergenic (TARDBPL-BMP2)* | 20p12.3 | colorectal | A | C | BioVU, MEC, WHI |
| RS910873 | *PIGU* | 20q11.22 | skin (melanoma) | A | G | BioVU, MEC, WHI |
| RS2284063 | *PLA2G6* | 22q13.1 | skin (melanoma) | A | G | BioVU, MEC, WHI |
| RS5945572 | *intergenic (CXorf67-NUDT11)* | Xp11.22 | prostate | A | G | BioVU, MEC, WHI |
| RS5945619 | *intergenic (NUDT11-TRNAE37P)* | Xp11.22 | prostate | C | T | BioVU, MEC, WHI |

***Genotyped in one or two studies (n=6***0)

| RS7538876 | *PADI6* | 1p36.13 | basal cell | A | G | MEC, WHI |
| --- | --- | --- | --- | --- | --- | --- |
| RS3790844 | *NRSA2* | 1q32.1 | pancreatic | T | C | WHI |
| RS2808630 | *CRPP1-CRP* | 1q23.2 | lung | C | T | MEC, WHI |
| RS801114 | *RHOU-ISCA1P2* | 1q42.13 | skin (basal cell) | G | T | MEC, WHI |
| RS721048 | *EHBP1* | 2p15 | prostate | A | G | MEC, WHI |
| RS1045485 | *CASP8* | 2q33.1 | breast | G | C | BioVU, WHI |
| RS6435862 | *BARD1* | 2q35 | neuroblastoma | G | T | MEC, WHI |
| RS189897 | *ITGA9* | 3p22.2 | nasopharyngeal | A | T | MEC, WHI |
| RS710521 | *TP63-LEPREL1* | 3q28 | bladder | A | G | MEC, WHI |
| RS1229984 | *ADH1B* | 4q23 | esophageal | C | T | MEC, WHI |
| RS4415084 | *FGF10-MRPS30* | 5p12 | breast | T | C | BioVU, WHI |
| RS2853676 | *TERT* | 5p15.33 | glioma | A | G | MEC, WHI |
| RS16886165 | *RPL26P19-MAP3K1* | 5q11.2 | breast | G | T | MEC, WHI |
| RS4624820 | *NDFIP1-SPRY4* | 5q31.3 | testicular | A | G | MEC, WHI |
| RS210138 | *BAK1* | 6p21.31 | testicular | G | A | MEC, WHI |
| RS3117582 | *BAT3* | 6p21.33 | lung | C | A | WHI |
| RS6939340 | *FLJ22536* | 6p22.3 | neuroblastoma | G | A | MEC, WHI |
| RS4132601 | *IKZF1* | 7p12.2 | cALL | C | A | MEC, WHI |
| RS7809758 | *DOC, FIGNL1* | 7p12.1 | cALL | G | A | MEC, WHI |
| RS12155172 | *SP8* | 7p15.3 | prostate | A | G | BioVU, MEC |
| RS157935 | *AC058791.1* | 7q32.3 | skin (basal cell) | T | G | MEC, WHI |
| RS12543663 | *intergenic (FAM84B-RP11-255B23.3)* | 8q24.21 | prostate | C | A | MEC, WHI |
| RS10086908 | *Intergenic (RP11-255B23.2)* | 8q24.21 | prostate | T | C | BioVU, MEC |
| RS1016343 | *intergenic (FAM84B-SRRM1P1)* | 8q24.21 | prostate | T | C | MEC, WHI |
| RS13252298 | *intergenic (FAM84B-SRRM1P1)* | 8q24.21 | prostate | A | G | MEC |
| RS6983561 | *RP11-255B23.3* | 8q24.21 | prostate | C | A | MEC, WHI |
| RS620861 | *RP11-255B23.3-POU5F1B* | 8q24.21 | prostate | G | A | BioVU, MEC |
| RS10090154 | *intergenic (POU5F1B)* | 8q24.21 | prostate | A | G | BioVU, MEC |
| RS9642880 | *POU5F1B-MYC* | 8q24.21 | bladder | T | G | MEC, WHI |
| RS10464870 | *intergenic (GSDMC)* | 8q24.21 | glioma | C | T | MEC, WHI |
| RS4295627 | *PVT1-GSDMC* | 8q24.21 | glioma | G | T | MEC, WHI |
| RS2294008 | *PSCA* | 8q24.3 | bladder, duodenal | T | C | MEC, WHI |
| RS2151280 | *CDKN2B* | 9p21.3 | skin (basal cell) | C | T | MEC, WHI |
| RS1412829 | *CDNK2A* | 9p21.3 | glioma, nasopharyngeal | C | T | MEC, WHI |
| RS4977756 | *CDKN2BAS* | 9p21.3 | glioma | G | A | MEC, WHI |
| RS10974944 | *JAK2* | 9p24.1 | myeloproliferative | G | C | MEC |
| RS965513 | *KRT18P13-FOXE1* | 9q22.33 | thyroid | A | G | MEC, WHI |
| RS505922 | *ABO* | 9q34.2 | pancreatic | C | T | MEC, WHI |
| RS10994982 | *AIRD5B* | 10q21.2 | cALL | A | G | MEC, WHI |
| RS10821936 | *ARID5B* | 10q21.2 | cALL | C | T | MEC, WHI |
| RS7089424 | *ARID5B* | 10q21.2 | cALL | C | A | MEC, WHI |
| RS2981579 | *FGFR2* | 10q26.13 | breast | T | C | MEC, WHI |
| RS11228565 | *intergenic (TPCN2-MYEOV)* | 11p13.3 | prostate | A | G | BioVU, WHI |
| RS7127900 | *TH-ASCL2* | 11p15.5 | prostate | A | G | MEC, WHI |
| RS11170164 | *KRT5* | 12q13.13 | skin (basal cell) | A | G | MEC |
| RS995030 | *KITLG* | 12q21.32 | testicular | G | A | MEC, WHI |
| RS671 | *ALDH2* | 12q24.12 | esophageal | A | G | MEC, WHI |
| RS2089222 | *MAP1LC3B2* | 12q24.22 | cALL | A | G | MEC, WHI |
| RS9543325 | *FABP5L1-KLF12* | 13q22.1 | pancreatic | C | T | WHI |
| RS2239633 | *CEBPE* | 14q11.2 | cALL | G | A | MEC, WHI |
| RS944289 | *BRMS1L-MBIP* | 14q13.3 | thyroid | T | C | MEC, WHI |
| RS10483813 | *intergenic (ZFP36L1)* | 14q24.1 | breast | T | A | MEC |
| RS999737 | *RAD51L1* | 14q24.1 | breast | C | T | BioVU, WHI |
| RS8034191 | *AGPHD1* | 15q25.1 | lung | C | T | MEC, WHI |
| RS6504950 | *COX11* | 17q22 | breast | G | A | BioVU, WHI |
| RS1859962 | *intergenic (CALM2P1-SOX9)* | 17q24.3 | prostate | G | T | MEC, WHI |
| RS8102476 | *DPF1-PPP1R14A* | 19q13.2 | prostate | C | T | BioVU, MEC |
| RS6010620 | *RTEL1* | 20q13.33 | glioma | G | A | MEC, WHI |
| RS4809324 | *RTEL1* | 20q13.33 | glioma | C | T | MEC, WHI |
| RS5759167 | *RPS25P10-BIK* | 22q13.2 | prostate | G | T | BioVU, MEC |

Table S3. Pleiotropic association of selected cancer susceptibility variants with the risk of common subtypes of non-Hodgkin lymphoma (NHL)

| Subtype  / SNP | Gene | GWAS | Risk (Ref.) Allele | BioVU | | MEC | | WHI | | Summary (meta-analysis) | | |
| --- | --- | --- | --- | --- | --- | --- | --- | --- | --- | --- | --- | --- |
|  |  |  |  | Cases /  Controls | OR (95% CI) | Cases /  Controls | OR (95% CI) | Cases /  Controls | OR (95% CI) | OR (95% CI) | *p-value*  (0.00044)† | Cochran Q  (*p-het*) |
| Follicular Lymphoma (FL) | | | | | | | | | | | | |
| rs11249433 | *EMBP1* | breast | C (T) | 71 / 8991 | 1.21 (0.87, 1.69) | 68 / 9014 | 0.99 (0.63, 1.54) | 150 / 5533 | 1.39 (1.09, 1.76) | 1.29 (1.08, 1.54) | 0.0095 | 1.84 (0.40) |
| rs6465657 | *LMTK2* | prostate | C (T) | 72 / 8987 | 0.69 (0.49, 0.97) | 68 / 9007 | 0.80 (0.54, 1.18) | 167 / 5942 | 0.89 (0.71, 1.12) | 0.82 (0.69, 0.97) | 0.0216 | 1.57 (0.46) |
| Diffuse Large B-Cell Lymphoma (DLBCL) | | | | | | | | | | | | |
| rs3131379 | *MSH5* | lung | T (C) | - | - | 90 / 9001 | 1.33 (0.69, 2.56) | 246 / 5945 | 1.42 (1.09, 1.85) | 1.41 (1.10, 1.80) | 0.0061 | 0.04 (0.85) |
| rs505922 | *ABO* | pancreatic | C (T) | - | - | 102 / 9011 | 0.83 (0.62, 1.13) | 245 / 5942 | 0.83 (0.68, 1.01) | 0.83 (0.71, 0.98) | 0.0301 | 0 (0.99) |
| rs7679673 | *RPL6P14-TET2* | prostate | C (A) | - | - | 102 / 8897 | 0.88 (0.36, 2.19) | 246 / 5935 | 0.84 (0.70, 1.01) | 0.84 (0.72, 0.98) | 0.0317 | 0 (0.98) |
| rs910873 | *PIGU* | melanoma | A (G) | - | - | 102 / 8970 | 0.88 (0.36, 2.19) | 246 / 5943 | 0.63 (0.42, 0.95) | 0.67 (0.46, 0.97) | 0.0351 | 0.43 (0.51) |
| Chronic Lymphocytic Leukemia (CLL) / Small Lymphocytic Lymphoma (SLL) | | | | | | | | | | | | |
| rs2735839 | *KLK3-KLK2* | prostate | G (A) | 35 / 8998 | 1.79 (0.81, 3.94) | 68 / 9016 | 1.52 (0.98, 2.36) | 61 / 5943 | 1.39 (0.80, 2.40) | 1.51 (1.10, 2.07) | 0.0099 | 0.27 (0.87) |
| rs13281615 | *8q24.21* | breast | T (C) | 35 / 8997 | 1.52 (0.91, 2.53) | 69 / 8989 | 1.43 (1.01, 2.01) | 61 / 5944 | 1.12 (0.78, 1.62) | 1.32 (1.05, 1.65) | 0.0158 | 1.25 (0.54) |
| rs189897 | *ITGA9* | naso-pharyngeal | A (T) | - | - | 69 / 8987 | 1.67 (1.10, 2.54) | 61 / 5942 | 1.12 (0.68, 1.83) | 1.41 (1.03, 1.94) | 0.0343 | 1.47 (0.23) |
| rs999737 | *RAD51L1* | breast | C (T) | 35 / 8923 | 2.09 (0.99, 4.39) | 69 / 8954 | 1.26 (0.75, 2.12) | 61 / 5919 | 1.31 (0.82, 2.09) | 1.40 (1.03, 1.92) | 0.0338 | 1.35 (0.51) |

* ORs and 95% CIs in individual studies were estimated in unconditional logistic regression models that were adjusted for age, sex (in BioVU and MEC) and ethnicity. Summary ORs and 95% CIs were estimated in a meta-analysis of fixed effects models.

† The *p-value* corrected for testing 113 SNPs is 4.4E-04.

Abbreviations: *p-het. (p*-values for heterogeneity across studies measured in Cochran’s Q statistic); BioVU (the biorepository of the Vanderbilt University), MEC (the Multiethnic Cohort Study), WHI (the Women’s Health Initiative).
